# Peripheral biomarkers and the philosophy of precision in treatment-resistant schizophrenia

**DOI:** 10.1016/j.nsa.2025.105523

**Published:** 2025-07-05

**Authors:** Stefan Jerotic

**Affiliations:** a Clinic for Psychiatry, University Clinical Centre of Serbia, Belgrade, Serbia; b Faculty of Medicine, University of Belgrade, Belgrade, Serbia

Pisanu and colleagues’ systematic review of peripheral biomarkers for treatment-resistant schizophrenia (TRS) (Pisanu et al., 2024) represents an ambitious attempt to synthesise an otherwise fragmented literature into a coherent narrative of genetic, epigenetic and immuno-metabolic correlates of antipsychotic non-response. By collating 75 studies that span genome-wide association analyses, methylomic screens, transcriptomic surveys and protein/metabolite assays, the authors provide what is arguably the broadest cartography to date of the biological terrain underlying TRS. Their principal conclusion–that evidence for any single class of markers remains modest and inconsistent–echoes the cautionary tone sounded by Aftab and Sharma (Aftab and Sharma, 2021) in their philosophical analysis of biomarker “pitfalls” in psychiatry, and invites a closer examination of how methodological and conceptual issues intersect.

## 1 Scope and methodological strengths

A major strength of the review is its panoramic scope. By juxtaposing common-variant polygenic risk scores (PRS) with rare disruptive variants, circulating cytokines with kynurenine-pathway metabolites, and gut microbiota with telomere attrition, Pisanu et al. make visible the sheer heterogeneity of TRS-related signals. Their decision to foreground genome-wide studies over candidate investigations corrects for historical publication bias and sets an empirical benchmark: despite sample sizes exceeding 10,000, the largest GWAS still reports only a 1–4 % SNP-based heritability for TRS and no single-nucleotide polymorphism that surpasses genome-wide significance (Pardiñas et al., 2022). This sober estimate serves as an antidote to biomarker hype and is fully consonant with Aftab's warning that diagnostic markers, even when statistically robust, may be “causally uninformative” and prone to reification.

## 2 Genomic findings and their interpretive limits

Pisanu et al. highlight a recurring but small elevation of schizophrenia-PRS among people who progress to clozapine, yet the effect seems contingent on urbanicity, age-at-onset and premorbid social adjustment. Such context dependence raises the question posed by Aftab: do biomarker associations merely redescribe the clinical construct that researchers have already defined, or do they carve nature at its joints? The authors acknowledge that clozapine prescription–a pragmatic proxy used in many of the reviewed studies–may conflate biological refractoriness with health-system variables (e.g., prescribing culture, delayed initiation). Future consortia could mitigate this by harmonising TRS phenotypes with the Treatment Response and Resistance in Psychosis (TRRIP) criteria and by embedding digital adherence metrics, thereby minimising pseudo-resistance.

## 3 Inflammation: signal or noise?

Of all protein biomarkers surveyed, interleukin-6 (IL-6) emerges as the most replicated correlate of TRS, accompanied by a broader T-helper-17 cytokine signature and elevated immune-inflammatory response system (IRS)/compensatory immune-regulatory system (CIRS) ratios. Yet here the confounding role of medication looms large. Antipsychotics (and clozapine in particular) exert pleiotropic effects on cytokine networks; Aftab reminds us that biomarker differences detected in medicated patients “cannot simply be assumed to be related to the disorder”. It has been noted that studies restricting analyses to drug-naïve or unmedicated individuals often attenuate inflammatory associations. A critical next step is therefore longitudinal sampling across the treatment continuum–baseline, trial failure, clozapine initiation–coupled with in-vitro pharmacodynamic controls, to disentangle trait from state.

## 4 Epigenetic and transcriptomic avenues

The review's synopsis of methylation and microRNA data is necessarily tentative: only four methylome studies and five transcriptomic studies satisfy inclusion criteria, and none converge on a common locus. Nonetheless, the demonstration that a six-probe methylation risk model discriminates TRS with up to 88 % accuracy in an independent Han-Chinese cohort is intriguing (Lu et al., 2023). Before such classifiers are heralded as “precision tools,” however, Aftab's caution against premature causal inference demands replication across ancestries and explicit modelling of environmental modulators (e.g., childhood trauma, metabolic syndrome). The field would benefit from integrative multi-omic designs – linking genetic liability, methylomic variation and cytokine profiles within the same individuals – to test whether TRS is best conceptualised as a systems-level dysregulation rather than an additive sum of disparate biomarkers.

## 5 Gut microbiota: a new frontier?

Only one study met the authors' eligibility criteria for gut microbiome analyses, identifying Porphyromonas over-representation and the absence of butyrate-producing Flintibacter in TRS (Manchia et al., 2021). While provocative, such ecological findings invite Aftab's reminder that biomarkers located “downstream” in causal chains may still be diagnostically useful without illuminating pathogenesis. Prospective interventional trials–prebiotic, probiotic or faecal microbiota transfer–could clarify whether dysbiosis is a driver, a passenger or an epiphenomenon of chronic antipsychotic exposure and dietary factors.

## 6 Integration versus reductionism

Perhaps the most compelling contribution of Pisanu et al. lies in their explicit call for “multi-omic” and systems-all-the-way-down approaches–an appeal that directly resonates with Aftab's advocacy of integrative models over linear bio-reductionism. Such a model resonates with notions of organizational causality (De Haan, 2020), in which mind–brain–environment systems are not hierarchically reducible, but co-constitutive. By framing biomarkers within a web of biological, psychological and social determinants, both papers converge on a shared epistemic humility: complex phenotypes like TRS are unlikely to yield a single diagnostic litmus test. Instead, composite panels that weight genetic burden, inflammatory tone, cognitive trajectories and environmental stressors may ultimately offer the most clinically actionable stratification.

## 7 Outstanding questions

Several questions arise from this synthesis. First, can genetic randomisation be leveraged to test whether IL-6 elevations are causally upstream of treatment resistance or merely correlates? Second, might polygenic risk scores that integrate metabolic-syndrome loci (given clozapine's cardiometabolic profile) outperform schizophrenia-PRS in predicting TRS onset? Third, how can future consortia ensure ancestrally diverse representation, given that most genomic samples reviewed are of European descent? Addressing these queries will not only refine biomarker validity but also enhance translational equity.

## 8 Conclusion

Pisanu et al. offer a meticulously curated atlas of peripheral biomarkers associated with treatment resistance in schizophrenia, while candidly acknowledging the field's present limitations: modest effect sizes, diagnostic heterogeneity, medication confounding and paucity of multi-layered datasets. When read alongside Aftab and Sharma's conceptual critique, the review underscores a pivotal lesson: biomarkers must be interpreted through a lens that marries statistical association with philosophical clarity about causation, construct validity and clinical utility. The path forward will require consortial harmonisation of TRS definitions, longitudinal and multi-omic sampling, and analytical frameworks that accommodate the recursive interplay of genes, brain, immune system and environment. Only then can the promise of precision psychiatry be reconciled with the complexity that both papers so carefully delineate.

## Conflicts of interest

None.
